# Proteasomal adaptations to FDA-approved proteasome inhibitors: a potential mechanism for drug resistance?

**DOI:** 10.20517/cdr.2021.27

**Published:** 2021-05-30

**Authors:** Kyung Bo Kim

**Affiliations:** Department of Pharmaceutics, College of Pharmacy, University of Kentucky, Lexington, KY 40536-0596, USA.

**Keywords:** Constitutive proteasome, immunoproteasome, carfilzomib, bortezomib, drug resistance

## Abstract

With proteasome inhibitors (PIs) becoming clinically available since 2003, outcomes for patients with multiple myeloma (MM) have dramatically changed, improving quality of life and survival. Despite the impressive treatment success, however, almost all MM patients who initially respond to these PIs eventually develop resistance. Furthermore, a portion of MM patients is inherently unresponsive to the PIs. Extensive mechanistic investigations identified several non-proteasomal signaling pathways suspected to be linked to the PI resistance, for which several excellent reviews are currently available. On the other hand, it is still unclear how cancer cells under high PI environments adapt to spare proteasome activity essential for survival and proliferation regardless of cancer evolution stages. This review outlines current progress towards understanding the proteasomal adaptations of cells in response to PI treatment to maintain necessary proteasome activity. A better understanding of cellular proteasomal changes in response to the PIs could provide a rationale to develop new therapeutics that could be used to overcome resistance to existing PI drugs.

## INTRODUCTION

One of the most fundamental processes in mammalian cells is the ubiquitin-proteasome system (UPS)- mediated protein degradation. In addition to disposing of misfolded or damaged proteins, the UPS is also responsible for the degradation of many signaling molecules in a highly controlled manner, regulating critical biological processes, such as cell cycle, inflammation, and DNA repair^[1]^. As such, the UPS is firmly believed to be a crucial component of several traditional cancer hallmarks, including angiogenesis, unlimited replication, and sustained proliferation^[2]^. The UPS’s fundamental importance to cancer cells is further endorsed by suggesting to include proteotoxic stress, which is delicately controlled by the UPS, as an additional cancer hallmark by Luo *et al.*^[3]^. More importantly, the UPS is also thought to be vital for cancer cells during their evolutionary processes to select clones conferring resistance to anticancer therapies, including proteasome inhibitor (PI)-based therapies^[4]^.

**Figure 1.**
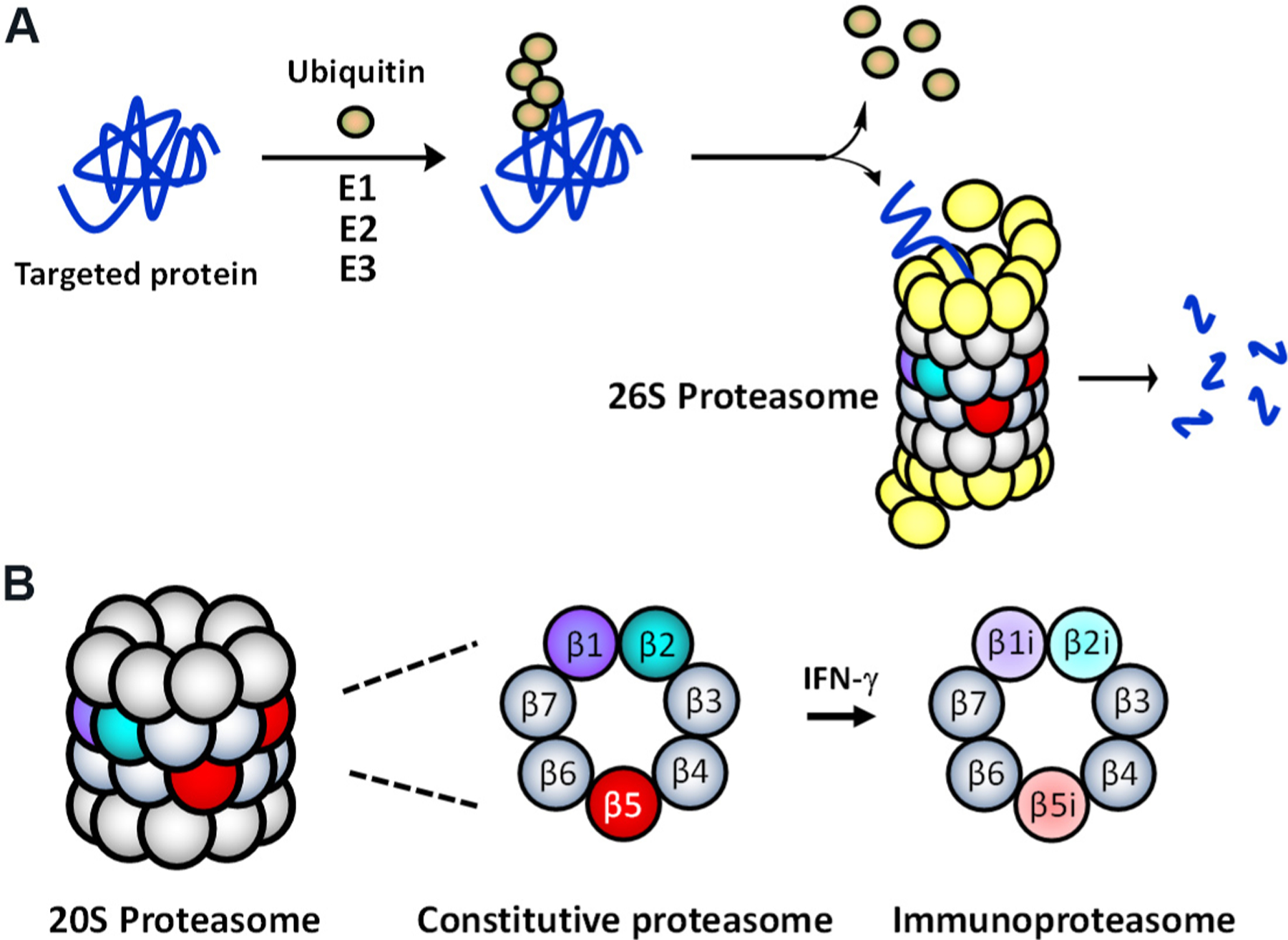
(A) The ubiquitin-mediated protein degradation pathway. Proteins, polyubiquitinated by an enzyme complex containing a ubiquitin-activating enzyme (E1), a ubiquitin-conjugating enzyme (E2), and a ubiquitin-protein ligase (E3), are recognized and degraded by the 26S proteasome. (B) Two main (or standard) 20S proteasome subtypes present in mammalian cells: the constitutive proteasome (cP) and immunoproteasome (iP).

The multi-protease complex 26S proteasome is a final executioner in the UPS, recognizing and deubiquitinating polyubiquitinated proteins and breaking them down into smaller peptide fragments [Figure 1A]. The 26S proteasome’s 20S core consists of 4-stacked rings comprising two identical outer α-rings and two identical inner β-rings, each containing 7 subunits [Figure 1B]. There are two main types of proteasomes in mammalian cells: the constitutive proteasome (cP) and immunoproteasome (iP). The cP has three catalytic subunits (β1, β2, β5) on each β-ring displaying three distinct substrate preferences: referred to as caspase-like (C-L), trypsin-like (T-L), and chymotrypsin-like (CT-L) activities, respectively. In immune cells, the constitutive subunits β1, β2, and β5, which are also commonly referred to as Y, Z, and X, are replaced by three immunosubunits β1i, β2i, and β5i, respectively, to form the iP. The three immunosubunits β1i, β2i, and β5i are also conveniently referred to as LMP2, MECL1, and LMP7. In non-immune cells, the iP can also be induced by pro-inflammatory cytokines such as interferon (IFN)-γ [Figure 1B]. Although the iP is shown to play a significant role in antigen presentation, its exact function in non-immune cells remains to be investigated.

**Figure 2.**
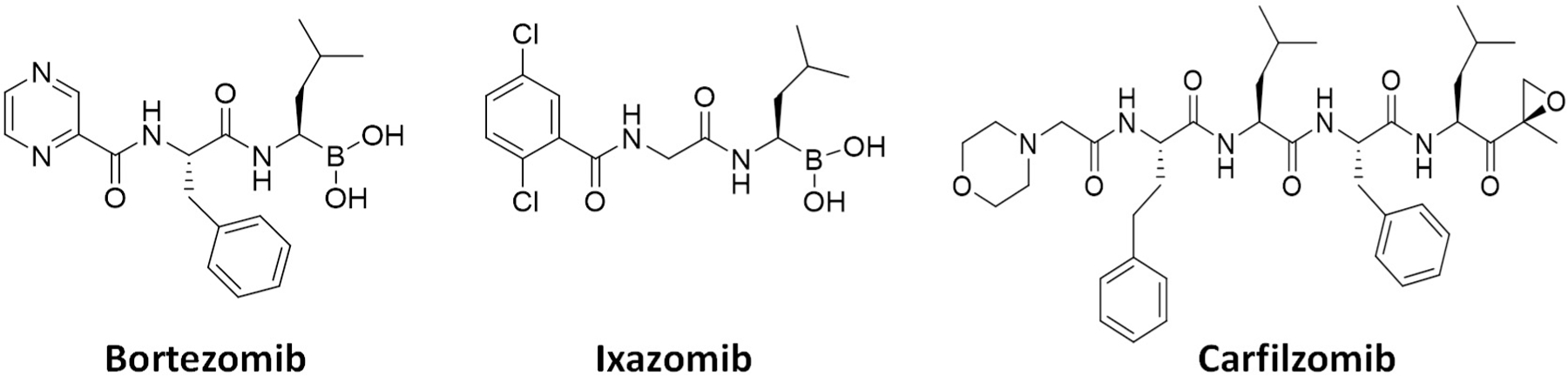
FDA-approved proteasome inhibitors. Bortezomib and ixazomib contain a boronic acid pharmacophore, while a tetrapeptide carfilzomib has an epoxyketone pharmacophore.

Due to increased proteotoxic stress and high metabolism in rapidly proliferating cancer cells, the demand for the UPS (and proteasome) activity is much higher than in resting normal cells, ultimately leading to the development of FDA-approved anticancer proteasome inhibitors (PIs)^[1]^. With several PIs becoming clinically available since 2003 [Figure 2], PI drugs, together with immunomodulatory agents, have become the mainstays of treatment regimens for multiple myeloma (MM) patients. The dipeptides bortezomib and its oral counterpart ixazomib share a boronic acid pharmacophore and bind to β5 via a tight non-covalent interaction between the boron atom and the nucleophilic oxygen lone pair of Thr01 within the β5 active site. On the other hand, the covalent inhibitor carfilzomib, a tetrapeptide with C-terminal α’,β’-epoxyketone, forms a seven-membered, 1,4-oxazepano adduct between the epoxyketone pharmacophore and the catalytic Thr01 within the β5 active site^[5,6]^. As a result, the covalent interaction of carfilzomib leads to less recovery of CT-L activity in cells than bortezomib^[7]^. Regardless, the prognosis of patients with MM has substantially improved over the years, increasing the 5-year survival rate from ~35% to ~54%^[8–10]^. Despite the treatment success, however, those who respond to PIs inevitably relapse^[11]^, and a portion of the MM patient population is also inherently unresponsive to the PIs^[12–14]^. Once relapsed on a PI drug, most patients are likely resistant to another PI drug, posing a major clinical hurdle. Clinical trials revealed that more than 75% of patients who relapsed on bortezomib-based therapies display cross-resistance to carfilzomib^[15]^, significantly restricting carfilzomib-based treatments.

Extensive mechanistic investigations identified several non-proteasomal PI resistance mechanisms, such as those involving dysregulations of unfolded protein response, autophagy, aggresomes, MARCKS proteins, or efflux transporters^[16–23]^. These mechanistic studies were performed primarily using established cell lines or small sample sizes of primary tissues, needing further verification for their clinical relevance and potential applicability to the development of new therapeutics to overcome PI resistance. On the other hand, it has been proposed that when MM cells in patients are exposed and adapted to therapeutic doses of PIs, the proteasome activity in MM cells might be sufficiently salvaged to allow for cell survival and proliferation^[24]^. This premise has been further bolstered by findings that cells with acquired or intrinsic PI resistance remain sensitive to proteasome activity disruption^[25–29]^, supporting that the proteasome activity is essential even to the PI-resistant cells. However, to date, it remains a controversial topic as to how cancer cells evade the inhibitory activity of PI to spare proteasome activity necessary for cell survival. Of note, it has been shown that PI-resistant cells are unlikely to have enhanced metabolic activity to inactivate PIs^[29]^. With this in mind, this review outlines the current understanding of proteasomal adaptations in response to PI treatment in cancer cells. It should be noted that the information summarized here is mainly from studies performed using cell line models and a limited number of primary samples. We expect that a better understanding of proteasomal adaptations to PIs in current clinical use could provide a strategy to develop new drugs that can be used to overcome clinical PI resistance.

## PROTEASOMAL ADAPTATIONS

### Point mutations in the PI-targeted proteasome subunits

Earlier studies performed using cell line models revealed that specific mutations occur primarily on a particular proteasome β-subunit, β5, which is responsible for the CT-L activity and a major target of all three FDA-approved PIs (bortezomib, carfilzomib, and ixazomib). For example, researchers observed a high level of acquired resistance to bortezomib when human myelomonocytic THP1 cells were exposed to increasing concentrations of bortezomib^[30]^. Further studies found a specific point mutation (*Ala49Thr*) residing in a highly conserved active site in the catalytic β5 subunit of the cP^[31]^. Given that Ala49 resides within the substrate-binding pocket, the Ala-to-Thr substitution is thought to impact bortezomib binding negatively. Similarly, using Jurkat cells cultured with increasing concentrations of bortezomib, another research group isolated two additional bortezomib-resistant clones containing β5 point mutation: Ala49Val and a conjoined mutant (Ala49Thr and Ala50Val), in addition to the previously reported Ala49Thr clone [Table 1]. These three mutants were ~22–67-fold more resistant to bortezomib compared to parental Jurkat cells. Modeling of β5 complexed with bortezomib suggests that both Ala49 and Ala50 are involved in interactions with bortezomib, verified by the X-ray study result^[32]^.

Multiple additional mutations were also identified in bortezomib-resistant blood cancer cell lines (AML, ALL, and MM), displaying ~40–170-fold resistance^[33]^. In these cell lines, mutations were clustered in the bortezomib binding region or its proximity. To distinguish between an expansion of a pre-existing β5 subunit mutant subclone and the *de novo* acquisition of a mutation and its subsequent outgrowth, researchers generated an additional independent panel of bortezomib-resistant cell lines. Identification of different mutations in these cell lines verified that the observed bortezomib resistance is due to *de novo* acquisition of multiple mutations in the β5 gene (*PSMB5*)^[33]^.

Allmeroth *et al.*^[34]^ also reported one previously known (*Ala49Val*) mutation and a new one (*Thr21Ala*) in KMS cell lines treated with increasing bortezomib concentrations [Table 1]. Interestingly, bortezomib-resistant KMS cells with Thr21Ala substitution were shown to be hypersensitive to carfilzomib and oprozomib. It is presumed that the replacement of Thr with Ala (having a smaller side chain) creates a bigger binding pocket, affording a tighter binding for carfilzomib and oprozomib, which possess a bulkier pharmacophore (epoxyketone) than bortezomib (boronic acid). In contrast, Ala49 to Val (having a bulkier side chain) mutation provided a cross-resistance to carfilzomib, presumably due to reduced accessibility of carfilzomib and oprozomib to the binding site within β5. Additionally, they employed an unbiased forward genetic approach in haploid cells using *N*-ethyl-*N*-nitrosourea mutagenesis to screen mutations that can impact the effectiveness of bortezomib, selecting several clones showing at least 2-fold bortezomib resistance. By analyzing these clones, they identified two new β5 mutation sites within the substrate pocket that can impact the effectiveness of bortezomib binding (Ser130, Tyr169). On the other hand, a systemic screening approach for bortezomib-resistant MM cell lines yielded 5 MM cell lines acquiring previously unidentified point mutations (*Met104Val* and *Thr80Ala*) [Table 1]^[35]^. Although these mutant-screening data may offer essential insights into the potential direction of second-generation PI treatment for patients who have developed resistance to initial PI treatment, their clinical relevance needs to be established.

For PIs other than bortezomib, Brünnert *et al.*^[36]^ reported the generation of MM cell lines that display a ~10-fold higher resistance to ixazomib than their parental cell lines. In two ixazomib-resistant MM cell lines, they found different β5 mutations (*Thr21Ala* in MM.1S and *Ala50Val* in L363 cell lines), whereas no mutations were found in the ixazomib-resistant AMO1 cell line. These ixazomib-resistant MM cell lines also exhibited high resistance to bortezomib and carfilzomib, indicating a common mechanical mode of resistance between the three PIs.

Until now, multiple large-scale screening efforts to identify somatic mutations of *PSMB5* from primary MM samples have failed to verify *PSMB5* mutations as a clinically relevant mechanism of PI resistance^[37–39]^. In a recent screening study with 1241 newly diagnosed MM patients, Barrio *et al.*^[40]^ identified a *Tyr42Cys* mutation of β5 from a single patient sample [Table 1]. Currently, the importance of the *Tyr42* mutation in clinical PI resistance remains unclear. They also reported four somatic *PSMB5* mutations from primary samples collected from a single MM patient refractory to bortezomib [Table 1]. Three of them (Ala20Thr, Ala27Pro, Met45Ile) reside within the substrate-binding site of β5, while Cys63Tyr is located near the S1 pocket area of the β5^[40]^. The Met45Ile mutant was previously reported in established cell lines with acquired bortezomib resistance^[41,42]^, and all the four mutations were shown to induce PI resistance, indicating that they are also involved with bortezomib binding as shown in structural and modeling studies^[7,41,42]^. While Ala20Thr and Ala27Pro mutations effectively abolished the effectiveness of bortezomib, both mutants remained sensitive to carfilzomib. Although a single patient sample was used to identify and verify these β5 mutants as a potential cause of PI resistance, the results suggest that PI resistance caused by specific β5 mutations can be overcome by alternative PIs that allow for stronger binding to the mutants. Ultimately, a larger scale of β5 mutation analysis using clinical samples may be needed to offer a helpful treatment direction in the future. There have been no reports showing β5 mutations as a potential cause of carfilzomib resistance in cell lines or primary tissue samples. Instead, several studies demonstrated that Pgp (P-glycoprotein) upregulation is a major mechanism of carfilzomib resistance in established cell lines^[19,43]^.

### Upregulation of β5 subunit, a primary target of proteasome inhibitor drugs

A few reports showed that *PSMB5* mRNA and β5 protein were overexpressed in response to chronic exposure to PIs. When Jurkat cells were repeatedly exposed to bortezomib over 6 months, researchers found increased expression of *PSMB5* mRNA, which was also coincident with increased CT-L activity [Table 2]^[44]^. In the same Jurkat cells, a considerable decrease in IκB-α levels was observed, indicating that the bortezomib-mediated upregulation of NF-κB activity may contribute to survival and proliferation of bortezomib-resistant cells. THP1 cells exposed to bortezomib over 6 months also led to bortezomib-resistant cells showing ~60-fold drug resistance, with a dramatic overexpression of β5 mutant (Ala49Thr) but not other subunits^[30]^. While increased β5 mutant expression led to no marked changes in the CT-L activity (measured using Suc-LLVY-AMC) compared to parental THP1 cells, siRNA-mediated PSMB5 silencing restored bortezomib sensitivity, indicating that the β5 mutant contributes to bortezomib resistance.

*PSMB5* mRNA overexpression has also been observed in bortezomib-resistant hepatocellular carcinoma (HCC) cell lines (HepG2 and Huh7) treated with stepwise increasing concentrations of bortezomib over ~6 months^[45]^. Bortezomib-resistant HepG2 cells displayed ~2.8 and ~6-fold higher CT-L and caspase-like (C-L) activities, respectively, relative to the parental cells, consistent with increased expression of β5 and β1 subunits in the drug-resistant cell lines [Table 2]. This observation suggests that the increased expression of β5 may be one of the mechanisms underlying bortezomib resistance regardless of cancer types. In addition, β5 was also highly upregulated in all three ixazomib-resistant MM cell lines examined (L363, MM.1S, AMO1), while β1 upregulation was found only in the ixazomib-resistant AMO1 cells^[36]^.

The overexpression of the *PSMB5* gene is also reported in primary MM cells collected from a refractory patient to bortezomib-based treatment^[46]^. Specifically, mRNA levels of PSMB5 in MM cells collected from patients who underwent 6-cycle of intensive bortezomib-based treatment were about 5-fold higher relative to before bortezomib treatment, with no *PSMB5* mutation [Table 2]. In a separate study using 10 MM patient samples collected before and after bortezomib treatment, researchers found that in 8 of those matched patient samples, PSMB5, 6, and 7 mRNA levels were significantly higher in bortezomib-treated primary samples than untreated counterparts^[35]^. It is currently unknown whether these upregulations observed in MM primary samples are linked to bortezomib resistance in these patients. Furthermore, PSMB5 mRNA was also highly expressed in primary triple-negative breast cancer (TNBC) tissue compared to normal tissues and non-TNBC breast cancer samples^[47]^. Not surprisingly, the CT-L activity in these primary TNBC tissues was considerably higher than in non-TNBC tissue breast cancer samples. Further analysis of survival data revealed that TNBC patients with relatively lower PSMB5 levels had much longer progression-free and overall survival than the high PSMB5 patients, suggesting the status of *PSMB5* expression as an important indicator of TNBC prognosis. Currently, it is unclear whether there is a correlation between *PSMB5* overexpression and drug resistance in TNBC. Mechanically, the overexpression of proteasome catalytic subunits in response to PIs is proposed to be related to a gain-of-function via increased assembly of 20S proteasome in response to PIs^[48]^.

Paradoxically, researchers found that reduced expression of 19S proteasome regulatory subunits is directly associated with intrinsic bortezomib resistance in cell line studies^[42,49]^. Furthermore, they also observed that reduced 19S subunit mRNA expression is correlated with bortezomib resistance and poor outcome for myeloma patients treated with bortezomib^[49]^. Similarly, Acosta-Alvear *et al.*^[50]^ identified several 19S subunits that induce resistance to carfilzomib in cancer cells using a systematic RNAi screening approach. In both cases, reduced expression of the 19S subunits is shown to cause significant changes in the spectrum of proteasome substrate and the remodeling of the transcriptome, indicating a potential mechanism of PI resistance. Interestingly, by analyzing gene expression data sets from 170 newly diagnosed, uniformly treated MM patients, Song *et al.*^[51]^ reported that the 19S subunit Rpn11, a deubiquitinating enzyme (DUB), is more highly expressed in patient MM cells than in normal plasma cells and its expression is directly correlated with poor patient survival. Although researchers show that inhibition of Rpn11 can overcome resistance to bortezomib, whether overexpression of Rpn11 is directly correlated with resistance to bortezomib is currently unknown.

### Aberrant expression of immunoproteasome and constitutive proteasome subunits

It has been shown that established MM cells and primary MM samples express iP subunits (β1i, β2i, or β5i)^[29,52]^, even though the presence of the fully assembled standard iP containing β1i-β2i-β5i in those cells has not been verified. Busse *et al.*^[53]^ also reported that three bortezomib-resistant cell lines, selected from 12 hematopoietic cancer cell lines by incubating with increasing concentrations of PS-341 (bortezomib), express commonly lower levels of both β2 and β1i than bortezomib-sensitive hematopoietic cancer cell lines examined. Specifically, bortezomib-resistant DG75 and KARPAS442 cell lines expressed much lower levels of β2i than bortezomib-resistant RAJI cells [Table 3], suggesting the impaired assembly of the standard 20S iP. They also found that solid cancer cell lines with low expression levels of β2 and iP subunits tend to show low bortezomib sensitivity^[53]^. In another study, Niewerth *et al.*^[23]^ reported that bortezomib-resistant sublines obtained from human leukemia cell lines (RPMI 8226, CCRF-CEM, and THP1) contain a significantly lower amount of iP subunits (in particular, β5i) while increased cP subunits (including β5 mutant) compared to their parental cell lines, perhaps promoting β5 mutant incorporation into the fully assembled 20S proteasome in place of β5i. Based on this finding, they suggested that downregulation of β5i expression may be a major determinant in acquiring bortezomib resistance. Similarly, bortezomib-adapted Namalwa (human Burkitt’s lymphoma) cells displayed increased expression of cP subunits (β1, β2, and β5) but a completely down-regulated expression of iP subunits (β1i, β2i, and β5i). Despite significantly increased expression of cP subunits at the expense of iP subunits, the proteasomal proteolytic activities were only slightly increased compared to the parental Namalwa cells.

There have been many clinical trials to evaluate the effectiveness of PIs in combination with other agents for the treatment of acute myeloid leukemia (AML) and acute lymphocytic leukemia (ALL), despite several setbacks showing only modest single-agent activity. Using samples from patients with relapsed and refractory acute leukemia, Niewerth *et al.*^[54]^ investigated the iP/cP expression ratios related to bortezomib-containing therapy responses. They found that AML patients who achieved complete remission (CR) have higher pre-treatment iP/cP ratios than patients who did not achieve CR. Similarly, ALL patients with higher iP/cP ratios showed an excellent initial response to the bortezomib-containing therapy; however, the result was not statistically significant due to insufficient sample sizes. Taken together, the result further supports that the iP/cP expression ratios may contribute to the bortezomib-containing therapy response in these disease types. Moving forward, further clinical studies may be needed to direct the course of the PI-containing therapy for the treatment of the diseases.

One of the major immunologic barriers in transplantation is donor-specific antibodies (DSAs), which have a deleterious effect on allografts. Bone marrow plasma cells (BMPCs) are a well-known source of long-term antibody production, causing severe transplantation rejection. In recent years, PIs, including carfilzomib, have been actively investigated as a promising strategy to suppress BMPC-mediated antibody production but suffer from acquired drug resistance of these BMPCs. Woodle *et al.*^[55]^ reported that BMPCs with acquired carfilzomib resistance display reduced sensitivity to other PIs such as bortezomib and ixazomib but enhanced sensitivity to the iP subunit β5i-selective inhibitor (ONX-0914). Using patient bone marrow biopsies, they confirmed that catalytically active iPs are upregulated in BMPCs collected after carfilzomib therapy^[55]^. Specifically, expression of β5i (and the proteasome activator PA28) was significantly higher in BMPC lysates collected after carfilzomib treatment than in those collected before. Expression levels of other proteasomal catalytic subunits (β1, β1i, β2, β2i, and β5) remained unchanged before and after carfilzomib treatment. The induction of β5i after carfilzomib therapy was also verified *ex vivo* by incubating carfilzomib in CD138+ BMPCs isolated from 8 individual HLA-sensitized patients. The incorporation of β5i into the catalytically active 20S proteasome complex was verified using a fluorescently labeled β5i-selective covalent probe, which fluorescently labels β5i in live cells. The data collectively support that upregulation of β51 contributes to acquired drug resistance of these BMPCs. However, it needs to be determined whether β5i upregulation leads to the assembly of the standard 20S iP containing β1i-β2i-β5i or non-standard 20S proteasome subtypes such as β1-β2-β5i or β1i-β2-β5i.

### Formation of non-standard 20S proteasomes

**Figure 3.**
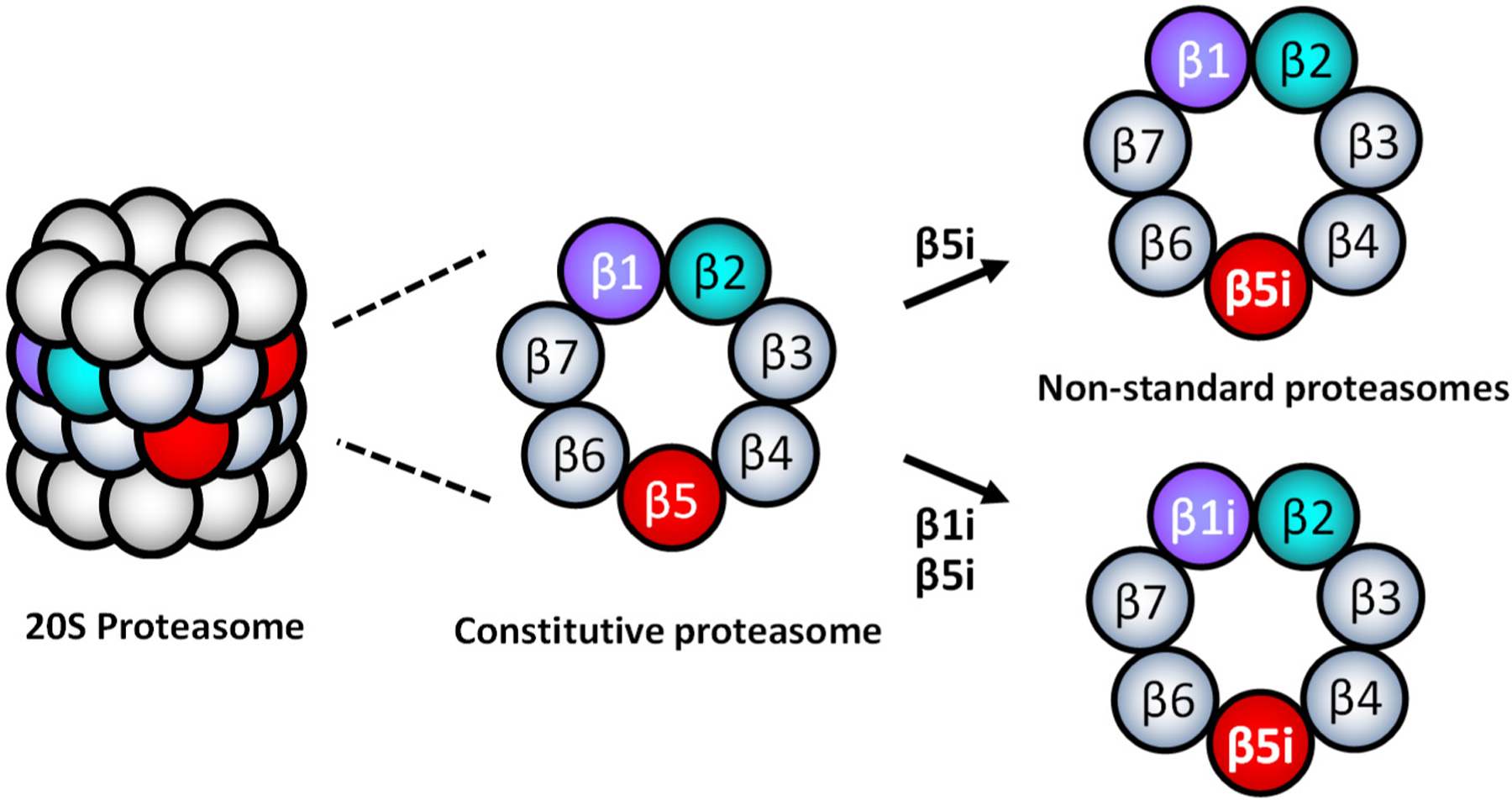
Examples of non-standard 20S proteasomes containing β5i-β1i-β2 or β5i-β1-β2 composition.

When the standard proteasome subtypes are present in cells, an equivalent expression of the standard sets of three catalytic subunits (β1-β2-β5 or β1i-β2i-β5i) is expected to be simultaneously detected. However, the evidence demonstrated that different tissue cells, including cancer, express inequivalent sets of standard catalytic subunits^[52,56–65]^, indicating the presence of non-standard types of 20S proteasomes containing mixed assortments of cP and iP catalytic subunits, such as β1-β2i-β5i or β1i-β2-β5. Parlati *et al.*^[66]^ reported that normal and malignant hematopoietic cells express high levels of β5 and β5i, while expression levels of β2i and β1i are significantly lower relative to β5i. Based on the subunit expression information, they proposed that non-standard 20S proteasomes containing β1-β2-β5i or β1i-β2-β5i are likely to present in the hematopoietically derived cells [Figure 3]^[66]^. While non-standard proteasome subtypes’ identity remains to be determined, non-standard proteasomes appear to display different sensitivities to PIs than cP and iP^[57,62,67,68]^. However, it is unclear whether the presence of non-standard proteasomes is linked to PI resistance.

In this regard, Lee *et al.*^[29]^ recently reported that intrinsically carfilzomib-resistant H727 cells express most of the iP and cP catalytic subunits while lacking β1i, suggesting the presence of non-standard proteasome subtypes such as β1-β2i-β5i, β1-β2i-β5, or β1-β2-β5i. Despite the *de novo* carfilzomib resistance, it has been shown that the proteasome activity in H727 cells remained vital for cell survival and proliferation. When incubated with IFN- to induce the assembly of the standard iP, H727 cells became sensitive to carfilzomib, suggesting that non-standard proteasome subtypes are involved with *de novo* carfilzomib resistance in H727 cells. It was also shown that alternative PIs with different structural features could overcome the *de novo* carfilzomib resistance in H727 cells^[69]^. Taken together, they suggested that non-standard proteasomes present in H727 cells may contribute to the intrinsic carfilzomib resistance, having a different drug binding affinity than the standard proteasome types.

## CONCLUSION

The proteasome-mediated protein degradation remains vital to cancer cells regardless of PI resistance status. Proteasomal adaptations in response to PI treatment are also noted in surviving and proliferating tumor cells. Point mutations or overexpression of proteasome catalytic β-subunits have been frequently observed in PI-resistant cell lines and limited numbers of primary tumor samples. Studies also suggest a potential role of non-standard 20S proteasome subtypes in PI resistance. However, the lack of extensive clinical evidence supporting the proteasomal contribution to acquired or intrinsic PI resistance indicates that there remains much room for further investigations of clinical samples. Moving forward, a better understanding of the proteasomal adaptation to PI treatment will help to determine a course of PI-based therapy. In summary, proteasomal adaptations appear to be partially responsible for PI resistance, either acquired or *de novo*, implying that the proteasome-targeting strategy may remain a valid therapeutic strategy for MM patients who relapsed on or refractory to PIs in current clinical use.

## Figures and Tables

**Table 1.** β5 mutations found in cells with acquired resistance to bortezomib

Cell lines	β5	Fold difference (cell viability)	Ref.
THP-1	*Ala49Thr*	~45–129	Oerlemans *et al.*^[30]^
Jurkat	*Ala49Thr*	Selected at 500 nM	Lu *et al.*^[31]^
Jurkat	*Ala49Thr* *Ala49Val* *Ala49Thr* & *Ala50Val*	~22 ~39 ~67	Lü *et al.*^[70]^
HT-29	*Cys63Phe*	~30	Suzuki *et al.*^[7]^
CEM	*Cys52Phe* *Ala49Val* & *Cys52Phe* *Ala49Thr*	~170	Franke *et al.*^[33]^
RPMI 8266	*Ala49Thr*	~40	
THP-1	*Ala49Thr* *Ala49Thr* & *Met45Ile* *Met45Ile*	Selected at 100 nM Selected at 500 nM Selected at 100 nM (2nd independent panel)	
Yeast	*Met45Ile* *Cys63Phe*	21.4 0.8	Huber *et al.*^[41]^
KBM7	*Met45Ile*	Exposed to 18 nM bortezomib and 700 nM MG132	Tsvetkov *et al.*^[42]^
RPMI 6226 KMS11R FR4R XG-1R	*Met104Val*	At least 5-fold	Shi *et al.*^[35]^
MM1.SR	*Thr80Ala*		
KMS-18 KMS-27	*Thr21Ala* *Ala49Val*	~2	Allmeroth *et al.*^[34]^
L363 MM.1S	*Ala50Val* *Thr21Ala*	9 Against ixazomib 13	Brünnert *et al.*^[36]^
Primary MM*	*Ala20Thr* *Ala27Pro* *Met45Ile* *Cys63Tyr*	From an MM patient refractory to bortezomib	Barrio *et al.*^[40]^
	*Tyr42Cys*	One out of 1,241 newly diagnosed patients with MM	

* Detected after initiation of PAD (bortezomib, A and Dex)-pomalidomide treatment at TP3. MM: Multiple myeloma.

**Table 2.** β5 overexpression found in cancer cells in response to bortezomib treatment

Cell lines	Overexpression	Fold difference	Ref.
Jurkat	PSMB5 mRNA (CT-L activity increased)	Exposed to bortezomib for 6 months	Lu *et al.*^[44]^
THP1	β5	~50 (β5 activity)	Oerlemans *et al.*^[30]^
HepG2 HuH7	β5, β1	~15 (cell viability) ~39 (cell viability)	Wu *et al.*^[45]^
U266 JJN3	β5 (& β6)	~2–8 (cell viability)	Shi *et al.*^[35]^
Primary MM	β5, β6, β6		
L363 MM.1S AMO1	β5, β1	9 Against ixazomib 13 11	Brünnert *et al.*^[36]^
Primary MM	PSMB5 mRNA	~5 (mRNA)	Shuqing *et al.*^[46]^
Primary TNBC	PSMB5 mRNA CT-L activity	Not determined (PSMB5 is indicative of poor prognosis)	Wei *et al.*^[47]^

**Table 3.** Aberrant expression of iP and cP subunits

Cell lines	Expression	Fold difference (cell viability)	Ref.
RAJI DG75 KARPAS442	↓ β2, β1i ↓ β2i (KARPAS442 & DG75 only)	Selected at 10–40 nM bortezomib	Busse *et al.*^[53]^
RPMI 8226 CCRF-CEM THP1	↓ β1i, β2i, β5i ↑ β1, β2, β5	~40–150	Niewerth *et al.*^[23]^
Namalwa (MES-SA)	↓ β1i, β2i, β5i ↑ β1, β2, β5	Growing at 12.5 nM of bortezomib	Fuchs *et al.*^[71]^
Primary bone marrow plasma cells	↑ β5i	After carfilzomib therapy	Woodle *et al.*^[55]^

↑: increased; ↓: decreased.
